# The Role of Indoleamine 2, 3-Dioxygenase in Immune Suppression and Autoimmunity

**DOI:** 10.3390/vaccines3030703

**Published:** 2015-09-10

**Authors:** Jacques C. Mbongue, Dequina A. Nicholas, Timothy W. Torrez, Nan-Sun Kim, Anthony F. Firek, William H.R. Langridge

**Affiliations:** 1 Center for Health Disparities and Molecular Medicine, Department of Basic Sciences, Loma Linda University School of Medicine, Loma Linda, CA 92354, USA; E-Mails: jmbongue@llu.edu (J.C.M.); dnicholas@llu.edu (D.A.N.); nkim@llu.edu (N.-S.K.); 2 California Baptist University, Riverside, CA 92504, USA; E-Mail: t2torrez722@gmail.com; 3 Department of Molecular Biology, Chonbuk National University, Jeon-Ju 54896, Korea; 4 Endocrinology Section, JL Pettis Memorial VA Medical Center, Loma Linda, CA 92357, USA; E-Mail: Anthony.firek@va.gov

**Keywords:** Indoleamine 2, 3-dioxygenase, Tryptophan, NF-κB, Vaccine, CTB-INS

## Abstract

Indoleamine 2, 3-dioxygenase (IDO) is the first and rate limiting catabolic enzyme in the degradation pathway of the essential amino acid tryptophan. By cleaving the aromatic indole ring of tryptophan, IDO initiates the production of a variety of tryptophan degradation products called “kynurenines” that are known to exert important immuno-regulatory functions. Because tryptophan must be supplied in the diet, regulation of tryptophan catabolism may exert profound effects by activating or inhibiting metabolism and immune responses. Important for survival, the regulation of IDO biosynthesis and its activity in cells of the immune system can critically alter their responses to immunological insults, such as infection, autoimmunity and cancer. In this review, we assess how IDO-mediated catabolism of tryptophan can modulate the immune system to arrest inflammation, suppress immunity to cancer and inhibit allergy, autoimmunity and the rejection of transplanted tissues. Finally, we examine how vaccines may enhance immune suppression of autoimmunity through the upregulation of IDO biosynthesis in human dendritic cells.

## 1. Introduction

**Figure 1:**
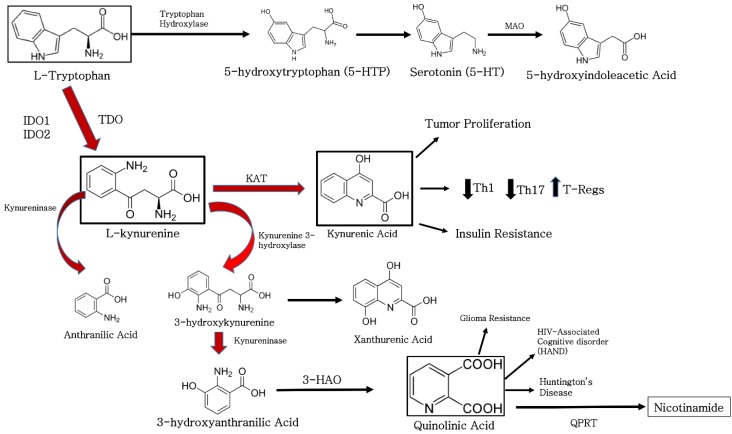
Pathways of tryptophan metabolism. Of the dietary tryptophan, 99% is metabolized via indoleamine 2, 3-dioxygenase (IDO) and tryptophan 2, 3-dioxygenase (TDO) to form kynurenine degradation products (red arrows). Additional enzymes in the pathway, kynurenine aminotransferase (KAT), monoamine oxidase (MAO), quinolinic-acid phosphoribosyl transferase (QPRT) and 3-hydroxyanthranilic acid oxidase (HAO), promote immune suppression through the inhibition of pro-inflammatory T-cells and induction of regulatory T-cell populations that stimulate pathologies as a result of insufficient or excessive immune suppression.

Indoleamine 2, 3-dioxygenase (IDO) is a mammalian cytosolic enzyme composed of two alpha-helical domains with a heme group located between them responsible for catalyzing the initial step in tryptophan catabolism via the kynurenine degradation pathway [1] (Figure 1). The first and rate-limiting step in this pathway is the conversion of tryptophan to N-formyl kynurenine, and until recently, this reaction was thought to be performed by either tryptophan 2, 3-dioxygenase (TDO) or indoleamine 2, 3-dioxygenase (IDO1) [2,3,4]. While TDO is widely distributed in both eukaryotes and bacteria [5], IDO1 is restricted to mammals and yeast [6]. A third tryptophan catabolic enzyme, named indoleamine 2, 3-dioxygenase-2, an indoleamine 2, 3-dioxygenase-like protein or “proto-indoleamine 2, 3-dioxygenase” (IDO2, INDOL1 or proto-IDO), was recently described and was found in mammals and in lower vertebrates [4,7]. Both IDO1 and IDO2 genes are conserved in mammals and are present in tandem on chromosome 8 [8,9]. Both IDO1 and IDO2 share significant identity at the amino acid level (43% for human and mouse proteins), but are structurally unrelated to the TDO enzyme protein. Expression of IDO2 is found in human DCs, but is not as ubiquitous as IDO1, although IDO2 mRNA can be detected in the liver, small intestine, spleen, placenta, thymus, lung, brain, kidney and colon [7]. The physiological role of IDO2 remains unclear, and unlike IDO1, its expression is not induced by virus infection or the presence of IFNγ [4]. Further, IDO2 is sensitive to inhibition by the D-isomer of 1-methyl tryptophan (D-1MT), a specific inhibitor of IDO [7]. The relevant sensitivity of IDO to inhibition lies predominantly in the putative effect of the D-isomer on suppression of cancer immune evasion. Interestingly, two non-synonymous single-nucleotide polymorphisms lie in the coding region of the IDO2 gene, both of which result in a loss of enzymatic activity [7]. This observation provides a basis for reducing the effect of IDO2 in cancer progression [2].

In this review, we will focus on the current knowledge of IDO1 biology and how IDO1 functions to inhibit activation of the human immune system. Indoleamine 2, 3-dioxygenase can act on multiple tryptophan substrates that include, L-tryptophan, 5-hydroxy-tryptophan, tryptamine and serotonin [10]. Through its expression in dendritic cells, monocytes and macrophages, IDO modulates T-cell behavior through catabolism of the essential amino acid tryptophan, which is obtained through the diet [11]. Through T-cell functions and other mechanisms to be described later, indoleamine 2, 3-dioxygenase is thought to play a role in a variety of pathophysiological processes that include antimicrobial and antitumor defense, neuropathology, immune-regulation, antioxidant activity and suppression of autoimmunity. Throughout this review, we will center our attention on the role of IDO1 in immunosuppression and experimental approaches that modulate IDO1 expression for the prevention and treatment of chronic inflammatory and autoimmune diseases.

## 2. The Function of Indoleamine 2, 3-Dioxygenase in Biological Systems

Indoleamine 2, 3-dioxygenase is a catabolic enzyme protein that functions to inhibit metabolism in a variety of biological systems that include mammalian reproduction, viruses, stem cells and the nervous system. The discovery of IDO function first occurred in mammals, which owe their continued existence to IDO-mediated immunosuppressive processes that prevent fetal rejection *in utero* [12]. Pioneering work by Munn, Mellor and their colleagues demonstrated that cells of the placenta express IDO1, which prevented maternal T-cell destruction of the fetus during pregnancy [12,13,14]. Arrest of tryptophan catabolism during pregnancy in mice enabled maternal T-cells to provoke fetal allograft rejection, confirming that placental cells synthesizing IDO1 can protect the mammalian fetus from maternal T-cell attack [15,16].

### 2.1. IDO Function in Stem Cells

Mesenchymal stem cells (MSCs) are multipotent stromal cells found in the bone marrow that differentiate into a wide variety of cell types that include osteoblasts (bone cells), chondrocytes (cartilage cells), myocytes (muscle cells) and adipocytes (fat cells). Mesenchymal stem cells provide a basis for improved tissue regeneration and gene therapy [17,18]. Although MSCs are mostly noted for their progenitor abilities, they also possess a broad immunological capacity. Earlier studies indicate that MSCs exert an immunosuppressive function in the human body [19]. In his studies, the author suggests MSCs do not have the innate ability to express IDO1, but gain this ability following stimulation by the pro-inflammatory cytokines interferon-γ (IFNγ) and tumor necrosis factor-α (TNFα) in combination with IL-1β [19]. To elucidate the molecular mechanisms underlying immunosuppression, MSCs from humans, monkeys and mice were compared, and considerable species variation in MSC-mediated immunosuppression was discovered. Mouse MSCs were shown to utilize nitric oxide (NO) as their immunosuppressive molecules, whereas human and monkey MSCs used IDO1 [20,21]. In humans, MSCs respond to pro-inflammatory cytokine production by synthesis of IDO1, which suppresses this inflammatory response, leading to immunological homeostasis [22]. This immunological tolerization response supports data suggesting that MSCs function as sensors of inflammation by adopting a pro-inflammatory or anti-inflammatory phenotype that modulates innate and adaptive immune responses *in vitro* and *in vivo* [23].

### 2.2. The Function of IDO in Cells of the Nervous System

In addition to establishment and maintenance of the blood-brain barrier, astrocytes in the central nervous system (CNS) play an important role as regulators of extracellular electrolyte and neurotransmitter balance. Together with microglia, astrocytes play a role as important modulators of CNS immune and inflammatory reactions [24]. The nervous system has its own self-contained, specialized form of immunity. Endothelial cells that make up the blood brain barrier catabolize L-tryptophan due to IDO1 stimulation of the kynurenine pathway [25]. T helper cells that express IFNγ can induce microglial cells to express IDO, which can initiate a negative feedback loop to suppress neural inflammation [26]. While IFN-γ signaling is needed to induce IDO in astrocytes, it was established recently that astrocytes express certain members of the toll-like receptor (TLR) family, in particular TLR3, the receptor for double-stranded RNA (dsRNA) [27,28,29]. Indoleamine 2, 3-dioxygenase was implicated in neurotoxicity and suppression of the antiviral T-cell response in HIV-generated encephalitis (HIVE) [27]. Hyeon-Sook Suh and his colleagues showed that the TLR3 ligand poly (I:C) (PIC) induces the expression of IDO in human astrocytes. PIC was found to be less potent than gamma interferon (IFN-γ), but more potent than IFN-β in inducing IDO1. PIC induction of IDO was shown to be mediated in part by IFN-β, but not IFN-γ, and both NF-κB and interferon regulatory factor 3 (IRF3) were also shown to be required [27]. These experimental results demonstrate that IDO1 can be induced by double-stranded RNA and suggests a therapeutic function for PIC in human viral infections.

Biosynthesis of IDO1 and the kynurenine pathway have been indicated as potential targets for neural degenerative disorders, as tryptophan degradation has been linked to the onset of neurological diseases, including Alzheimer’s disease, Huntington disease and even psychological depression [26]. In the brain, IDO1 can be induced in microglia by interferon-gamma-producing T helper 1 (Th1) cells, thereby initiating a negative feedback loop, which can down-modulate neuro-inflammation in experimental autoimmune encephalomyelitis (EAE), the animal model of multiple sclerosis (MS). This protective effect could be counteracted by the production of neurotoxic metabolites of the kynurenine pathway, such as quinolinic acid, which is produced upon IDO induction. Some metabolites of the kynurenine pathway can pass the blood-brain barrier and may act as neurotoxins during systemic infection. Two tryptophan degradation products, quinolinic acid (QUIN) and 3-hydroxyanthranilic acid (3-HAA), exhibit neurotoxic properties [26]. QUIN is an endogen *N*-methyl-D-aspartate (NMDA) receptor agonist. At micromolar concentrations, the cytotoxic effect of QUIN can be mimicked in primary cortical neuronal cell cultures [30]. The second neurotoxic Trp metabolite is 3-HAA, which is unstable under physiological conditions. Upon spontaneous auto-oxidation, 3-HAA produces reactive radical species, which, in turn, induce oxidative stress and apoptosis in neurons [31]. These data suggest IDO1 may act as a double-edge-sword in the nervous system.

## 3. Mechanisms of IDO1 Induction and Function

Sustained access to nutrients is a fundamental metabolic requirement for prokaryotic and eukaryotic cell maintenance and proliferation. Controlling the supply of available nutrients is an ancient strategy for the regulation of cellular responses to stimuli. Aside from its role as one of the limiting essential amino acids in protein metabolism, tryptophan (TRP) serves as a precursor for the synthesis of the neurotransmitters serotonin and tryptamine, as well as for the synthesis of the anti-pellagra vitamin nicotinic acid and the hormone melatonin [32]. By involvement in a variety of metabolic pathways, TRP and its metabolites regulate neurobehavioral effects that include appetite, the sleeping-waking-rhythm and pain perception. TRP is the only amino acid that binds high levels of serum albumin [32,33]. Through IDO degradation of tryptophan, cells that express the enzyme mediate potent effects on metabolic events responsible for innate and adaptive immune responses to inflammatory insults. In addition, IDO1 was shown to alter immune responses through a variety of mechanisms dependent on the regulation of cell metabolism. In the sections that follow, we will identify mechanisms by which IDO1 activation was shown to modulate eukaryotic cell functions leading to stimulation or suppression of the diseased state.

### 3.1. Signaling Pathways Responsible for the Induction of IDO1 Expression

Indoleamine 2, 3-dioxygenase is not constitutively expressed in cell systems. Rather, various stimuli and signaling pathways induce transcription and translation of metabolically-active IDO1 enzyme protein. Various transcription factors were shown to regulate the expression of IDO1 [34,35]. The IDO1 promoter contains nucleotide sequences that allow regulation through interferon sequence response-like elements (ISRE), GAS (palindromic gamma-activated sequences) and non-canonical NF-κB (nuclear factor kappa-light-chain-enhancer of activated B-cells) consensus sequences [36,37,38]. Mutation or deletion of portions of two ISRE cis-acting (ISRE1 and ISRE2) response elements resulted in decreased IDO1 expression levels [39]. Deletion of ISRE1 decreased the ability of IFN-γ to induce IDO1 by 50-fold, and point mutations at two alanine residues of ISRE2 at −111 decreased the ability of IFN-γ to induce IDO1 by four-fold [40]. The distance between these response elements does not influence IDO1 expression, as the deletion of 748 bps between the elements had no effect on IDO1 synthesis [40]. In addition to the ISRE elements, a nucleotide sequence with a partial homology to the IFN-gamma-responsive sequence (GAS) was shown to be located in the promoter region of the IDO1 gene [39]. In murine 3B6A cells, a cell line with a defect in IDO1 activity, Stat 1α was shown to bind to GAS and to restore IDO1 induction [36]. The consensus sequence PuGGAGAPyTTPu is required for non-canonical binding of NF-κB [41]. The IDO1 promoter contains three partial RelB/p52 binding sites: AGGAGACACA, GGGAGACAGA and AGGAGAAAGA around position −2000 [41,42]. Manches *et al*. demonstrated by luciferase assay and ChIP analysis experiments that RelB bound directly to non-canonical NF-κB binding sites in the promoter regions of mammalian DNA and drives IDO1 gene expression [43,44]. 

**Figure 2:**
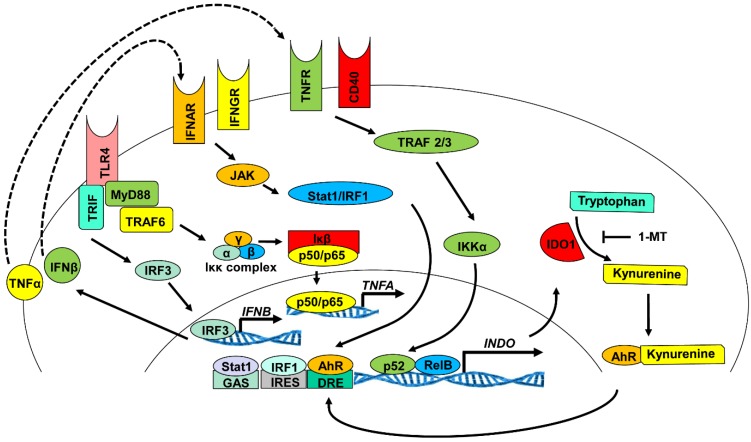
Mechanism of IDO1 induction in dendritic cells: transcription and translation. Several molecular stimuli and signaling pathways were shown to induce the transcription and translation of metabolically-active IDO1 enzyme [35,40,50]. The IDO1 promoter contains nucleotide sequences that allow regulation of transcription through interferon sequence response-like element (ISRE) upstream consensus sequences, GAS (palindromic gamma-activated sequences) and non-canonical nuclear factor kappa-light-chain-enhancer of activated B-cells (NF-κB) [42,50]. In addition, the IDO1 promoter region contains three partial non-canonical RelB/p52 binding sites: AGGAGACACA, GGGAGACAGA and AGGAGAAAGA located near position −2000, which is located downstream of NF-κB-driven IDO upregulation following stimulation of the TLR4, INFGR, IFNAR, TNFR and CD40R signaling pathways [43].

There are several receptor/ligand signaling pathways upstream of these transcription factors that can regulate IDO expression. Toll-like receptors (TLRs), tumor necrosis factor superfamily members (TNFRs), interferon beta receptor (IFNBR), the interferon gamma receptor (IFNGR), transforming growth factor beta receptors (TGFBRs) and the aryl hydrocarbon receptor (AhR) all can activate signaling mechanisms that either induce or maintain IDO1 expression. Stimulation of TLR3 and TLR4 was shown to induce IDO1 production in dendritic cells, while TLR7/8 was shown to upregulate IDO1 in monocytes [42,43]. The mechanism by which TLR4 ligation activates IDO1 expression was shown to contribute to autocrine signaling from TNF-α and/or IFN-β [45] (Figure 2). For example, LPS induction of IDO1 in a monocyte cell line is decreased when TNF-α is blocked with neutralizing antibodies [46]. Ligation of TLR4 activates both MyD88-dependent and MyD88-independent adaptor-driven signaling pathways, which leads to the activation of canonical NF-κB and IRF3 transcription factors. Activation of NF-κB leads to the expression of the pro-inflammatory cytokine TNF-α, while the transcription factor IRF3 operating in conjunction with NF-κB induces the pro-inflammatory cytokine IFN-β. These cytokines bind their receptors (TNFR and IFNAR) to activate the non-canonical NF-κB and JAK-STAT signaling pathways, which leads to the transcription and translation of IDO protein [47].

**Figure 3:**
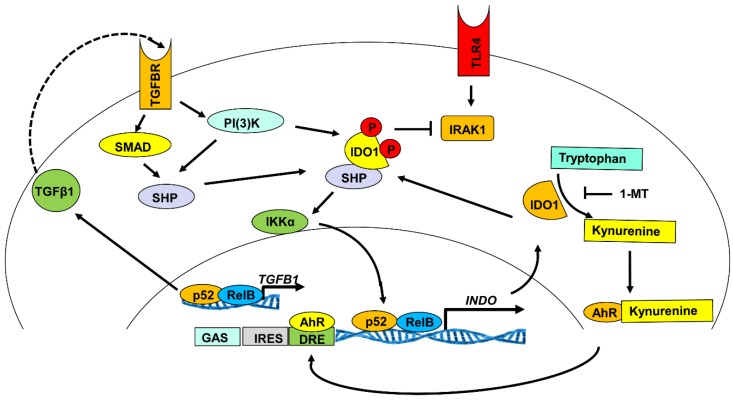
The TGF-β-IDO-SHP axis. The TGF-β-IDO-SHP axis activates the non-canonical NF-κB pathway [49]. In splenocytes, SHP-1 inhibits the protein kinase IRAK1 and tips the balance of activation of the canonical *versus* non-canonical NF-κB signaling pathway in favor of the latter, resulting in upregulated production of type I interferon [49]. In addition to TGF-β, the aryl hydrocarbon receptor (AhR) has also been shown to play a role in IDO production [51,53]. Interestingly, kynurenines produced from IDO’s enzymatic activity can also bind and activate as an endogenous ligand, AhR [53,54]. Abbreviations: IFN (α β, γ,): interferon alpha, beta, gamma; TGF-β: transforming growth factor-beta; TNF: tumor necrosis factor; TLR4: toll-like receptor 4; TRAF: TNF-receptor associated factors; TRIF: TIR-domain-containing adapter-inducing interferon-β; SHP: orphan nuclear receptor small heterodimer partner; SMAD: extracellular signal transducers from TGF-β ligands to the nucleus; IRF (1, 3): and interferon regulatory factor 1,3.

The ligation of IFNGR by IFN-γ is another established pathway known to stimulate production of IDO in immune cells. In human monocyte-derived DCs, stimulation with IFN-γ induces IDO through the JAK/STAT signaling pathway [45,47,48]. Of greater interest is the regulation of IDO by TGF-β. Unlike the previous signaling mechanisms, TGF-β was shown to produce delayed expression of IDO in plasmacytoid dendritic cells (pDCs) that is long lasting and stable [49]. Upon binding to the TGF-beta receptor (TGFBR), both the Smad-dependent and Smad-independent pathways (phosphatidylinositol-3-OH kinase (PI(3)K) are activated and signal the induction of non-canonical NF-κB pathway upregulation of IDO biosynthesis [50]. Upon stimulation of IDO1 biosynthesis, TGF-β generates a positive feedback loop for sustained production of TGF-β and IDO1 through a PI (3) K-dependent mechanism [49]. This signaling mechanism relies on the ability of TGF-β to activate the signaling capability of IDO1, which is described in detail in the following sections. IDO1 signaling mediated by TGF-β promotes transcription and translation of more TGF-β, which, in turn, continues to upregulate IDO1 production (Figure 3).

In addition to TGF-β, the aryl hydrocarbon receptor (AhR) was also shown to play a role in IDO production. In mouse bone marrow-derived DCs, Nguyen *et al.* demonstrated that AhR^−/−^ DCs do not produce IDO following LPS or CpG treatment [51]. This result suggests that AhR may be necessary for TLR4 and TLR9 induction of IDO in DCs. Secondly, kynurenines produced in response to IDO’s enzymatic activity can bind and activate AhR as an endogenous ligand. (Figure 3). Vogel *et al.* demonstrated that AhR can partner with RelB to associate with DNA by binding the IDO1 promoter at putative dendritic cell responses element (DRE) consensus sequences and, thus, promote AhR-dependent induction of IDO1 [52,53].

Based on the observation that CD40 ligand (CD40L) induces IDO1 biosynthesis through non-canonical NF-κB signaling in human DCs [35], NF-κB signaling pathway involvement in cholera toxin B subunit-proinsulin fusion protein (CTB-INS)-induced IDO1 biosynthesis was assessed in a study conducted in our laboratory. In this study, NF-κB activation for vaccine upregulation of IDO1 was identified with the help of two specific NF-κB pharmacological inhibitors, 2-Amino-6-[2-(cyclopropylmethoxy)-6-hydroxyphenyl]-4-(4-piperidinyl)-3-pyridinecarbonitrile (ACHP) and dehydroxymethylepoxyquinomicin (DHMEQ) [34]. 

Although there are various mechanisms known to promote IDO induction, the function of IDO remains consistent, to promote overall immune suppression, as well as control of some infectious pathogens. In response to inflammatory stimuli, IDO functions as an immune regulator to keep pro-inflammatory signaling in check. Conversely, IDO is important, but not essential for the maintenance of immune tolerance, as IDO^−/−^ mice do not die from autoimmunity [14]. The mechanisms by which IDO exerts its immunosuppressive effects are discussed in the following sections.

### 3.2. Enzymatic Activity of IDO1

Indoleamine 2, 3-dioxygenase was shown to inhibit DC maturation through tryptophan starvation via a generalized reduction in cellular energetics and through the generation of secreted kynurenines known to effectively stimulate pro-inflammatory T-cell apoptosis [34]. Additional experimental findings showed in addition to tryptophan depletion, paracrine effects of secreted kynurenine tryptophan degradation products may contribute to DC tolerogenesis through increased recruitment of regulatory T-cells [55,56,57]. The immunosuppressive activity of IDO was first speculated to be solely a function of the physical depletion of tryptophan from the intracellular environment, thus starving the metabolism of DCs, T-cells and other effector cells of the immune system. Tryptophan depletion is sensed in eukaryotic cells through activation of the general control non-repressed 2 (GCN2) kinase, which directly binds uncharged tRNAs [14,58,59]. Tryptophan depletion was shown to induce the GCN2 pathway, to downregulate the CD3 ζ-chain in CD8^+^ T-cells and to inhibit Th17 cell differentiation [14,55]. Additionally, in a recent study conducted by Chaudhary and colleague, antibody-mediated inflammatory kidney injury and renal disease in a mouse nephrotoxic serum nephritis model was inhibited by amino acid metabolism and a protective autophagic response. The metabolic signal was driven by IFN-γ-mediated induction of indoleamine 2, 3-dioxygenase 1 (IDO1) enzyme activity with subsequent activation of a stress response dependent on GCN2. These findings outline the IDO-GCN2 pathway in glomerular stromal cells as a critical negative feedback mechanism that limits inflammatory renal pathologic changes by inducing autophagy [60]. Recent work provided definitive evidence of an important role for kynurenine metabolites in IDO-mediated modulation of immune function [11,56,61]. Further, these studies also demonstrated IDO-dependent apoptosis of thymocytes and terminally-differentiated antigen-specific CD4^+^ T-cells [15,62]. Previous work showed that transgenic DCs with high levels of IDO expression and tryptophan metabolites (*i.e.*, l-kynurenine, 3-hydroxykynurenine and 3-hydroxyanthranilic acid) were able to irreversibly suppress allogeneic T-cell proliferation *in vitro* [15,55,63] (Figure 1). In these studies, immuno-suppressive tryptophan catabolites were shown to exert a cytotoxic action on CD3^+^ cells. This action preferentially affected activated T-cells and gradually increased with exposure time. In addition to T-cells, B-cells and natural killer (NK) cells were also killed while DCs remained unaffected [63]. Similar results were obtained in another study where three tryptophan catabolites (*i.e.*, l-kynurenine, picolinic acid and quinolinic acid) were shown to be responsible for IDO-induced inhibition of T- and NK-cell proliferation potentiated by tryptophan depletion [15,64].

### 3.3. Indoleamine 2, 3-Dioxygenase 1 Signaling Activity

The immunosuppressive effect of IDO was recently shown in non-obese diabetic (NOD) mice to require both enzymatic and signaling functions [49,65]. Treatment of mouse plasmacytoid DCs with transforming growth factor-β (TGF-β) conferred regulatory effects on IDO1 that were shown to be mechanistically separable from its enzymatic activity [11,49,66]. The TGF-β-IDO axis was found to mediate durable regulatory functions, resulting in the generation and maintenance of regulatory T-cell populations [49,61]. In these studies conducted by Pallotta *et al.*, IDO signaling activity was triggered in plasmacytoid dendritic cells (pDCs) by transforming growth factor-β (TGF-β) through the non-canonical NF-κB pathway, resulting in the induction of long-lasting IDO expression and autocrine TGF-β production in a positive feedback loop [49]. In addition, IDO was found to be involved in intracellular signaling events responsible for self-amplification and maintenance of a stable regulatory pDC phenotype (Figure 2). Additionally, CpG oligodeoxynucleotides (CpG-ODNs) known to stimulate innate and adaptive immunity by binding to TLR9 molecules [67], induced selective IDO1 expression by a minor population of splenic CD19^+^ dendritic cells (DCs) that did not express the plasmacytoid DC marker 120G8. Following CpG-ODN treatment, CD19^+^ DCs acquired potent IDO-dependent T-cell suppressive functions. Signaling through IFN type I receptors was essential for IDO upregulation, and CpG-ODNs induced selective activation of STAT-1 in CD19^+^ DCs [68]. In the same line, a discrete population of splenocytes with attributes of dendritic cells (DCs) and co-expressing the B-cell marker CD19 is uniquely competent to express the T-cell regulatory enzyme indoleamine 2, 3-dioxygenase (IDO) in mice treated with TLR9 ligands (CpGs) [69]. Johnson and colleagues have shown that IDO-competent cells express the B lineage commitment factor Pax5 and surface immunoglobulins and that CD19 ablation abrogated IDO-dependent T-cell suppression by DCs [69]. This study has shown that IDO-competent cells constitute a distinctive B-lymphoid cell type with quintessential T-cell regulatory attributes and phenotypic features of both B-cells and DCs.

The aryl hydrocarbon receptor (AhR) was shown to cause immune suppression after binding dioxin [70]. The aryl hydrocarbon receptor may be central to naive T-cell differentiation into Foxp3^+^ regulatory T-cells (Tregs) rather than pro-inflammatory Th17 lymphocytes [71]. In this study performed by Mezrich and his colleagues, kynurenines were shown to activate AhR, leading to AhR-dependent Treg generation. Together, the above studies reinforce the involvement of IDO in the generation of Tregs, as well as highlighting the central importance of IDO’s signaling capabilities [72,73,74].

## 4. The Role of IDO in Immune Suppression

### 4.1. The Function of IDO in Organ and Tissue Graft Survival

Acute and chronic graft rejection during solid organ and tissue transplantation is a demanding challenge for surgeons and patients. Current treatments employ a general immunosuppressive regimen, which leaves the patient vulnerable to common pathogens, and immuno-suppressive therapy usually must be administered lifelong with potentially severe side effects [75,76]. *In vivo* experiments have shown that *IDO1* gene knockout mice experience acute rejection of transplanted MHC mismatched grafts, while wild-type mice with high tryptophan catabolism experienced long-term graft survival [77]. Further experiments have shown that the dendritic cell costimulatory factor CD83 (sCD83) induced long-term IDO expression in DCs via upregulation of TGF-β both *in vitro* and *in vivo*, resulting in the induction of a long-lasting allograft tolerance in combination with a locally-restricted immunosuppressive environment [78]. Another study showed that IDO-mediated tryptophan degradation in renal allograft recipients is increased both before and during allograft rejection [79]. This result suggests that promotion of IDO1 biosynthesis and activity might have significant implications for immune suppression of tissue rejection in transplantation biology that extend far beyond the application of IDO as a possible diagnostic tool for the detection of acute allograft rejection. Additionally, inhibition of CD8^+^ T-cell-mediated cytotoxic function was found to be an important mechanism behind IDO’s immune-modulating property. In a study conducted by Liu *et al.*, in an experimental rat lung allograft, enhanced IDO activity was achieved by using a lung-tissue-targeted non-viral human IDO1 gene transfer approach, which reduced, but did not eliminate, infiltrating CD8^+^ T-cells. The impaired cytotoxic function seen in the IDO-treated CD8^+^ T-cells was accompanied by defects in the production of granule cytotoxic proteins, including perforin and granzyme A and B [79,80].

### 4.2. The Function of Indoleamine 2, 3-Dioxygenase in Viral Infection

Though the role of IDO in many viral infection models is presently unclear, some viruses can create an advantage for their replication by stimulating the enzyme’s catabolic activity to suppress unwanted immune responses in mammalian cells. Specifically, human immune deficiency virus (HIV) and Epstein-Barr virus are two well-known virus examples that increase cellular levels of IDO during infection [81,82,83]. It has been suggested that HIV may induce IDO expression to inactivate the human immune system. HIV is a lentivirus (retrovirus subgroup) that infects CD4^+^ T-cells, macrophages and dendritic cells [84,85]. Facilitating the spread of HIV infection, the virus evades the direct killing mechanisms of CD8^+^ cytotoxic lymphocytes that recognize HIV-infected cells by inducing IDO synthesis [86]. Earlier studies show that HIV stimulates IDO biosynthesis to block the function of pro-inflammatory CD4^+^ T helper cells and to stimulate immunosuppressive Treg cell responses [83]. A recent report showed that IDO1 was overexpressed in lymphoid tissues during HIV infection [83]. Further, increased tryptophan catabolism, measured as an increase in the kynurenine/Trp ratio, was shown to occur in HIV-infected patients [87]. Together, these data suggest that HIV depends on the immunosuppressive properties of IDO to facilitate the immune evasion processes.

The Epstein–Barr virus (EBV), also referred to as human herpesvirus 4 (HHV-4), is one of eight virus strains in the herpes virus family and is one of the most common human pathogenic viruses. The herpesvirus 4 strain is best known as the cause of infectious mononucleosis (glandular fever) [88]. This virus strain was also shown to be associated with specific forms of cancer, including Hodgkin’s lymphoma, Burkitt’s lymphoma, nasopharyngeal carcinoma and HIV-associated conditions, including hairy leukoplakia and central nervous system lymphomas [88,89]. The EB virus is known to infect monocytes/macrophages, intraepithelial macrophages and Langerhans dendritic cells [90,91]. Infection of monocytes with EBV was shown to suppress their phagocytic and antiviral activity [92,93]. More recently, EBV infection was shown to induce IDO mRNA, protein and enzymatic activity in human monocyte-derived macrophages (MDMs) [81]. This important finding suggests that EBV-mediated IDO expression in nasopharyngeal carcinoma tumor stroma may provide an immune-suppressed T-cell microenvironment that facilitates virus infection.

### 4.3. The Role of Indoleamine 2, 3-Dioxygenase in the Promotion of Cancer Cell Survival

While escape from the immune response is essential for cancer progression, mechanisms underlying this process remain unclear. The catabolism of tryptophan in tumor cells mediated by IDO1 has been increasingly identified as a critical micro-environmental factor involved in aiding immune escape through suppression of anti-tumor immunity [94,95]. Stimulation of the tryptophan catabolic pathway was shown to create an immuno-suppressive milieu in tumors and in tumor-draining lymph nodes through accumulation and secretion of immunosuppressive tryptophan catabolites that lead to induction of T-cell anergy, apoptosis and increased proliferation of immunosuppressive regulatory T-cells (Tregs) [96]. Thus, IDO is capable of biasing the immune system towards tumor support by decreasing the level of pathogenic inflammation in the tissue microenvironment surrounding the tumor. Clinically, studies of ovarian, endometrial and colorectal cancer have shown that increased expression of IDO1 was associated with poor survival outcomes [96]. Based on the enzyme’s immunosuppressive functions, IDO1 is becoming established as a target for drug discovery in cancer immunotherapy [95,97]. Human primary gastric, colon and renal cell carcinomas were shown to constitutively express both IDO1 and IDO2 mRNA, whereas cancer cell lines generally required induction of IDO by interferon-gamma (IFNγ) [8]. In this study, treatment of HeLa cells with IDO1 siRNA resulted in the prevention of tryptophan degradation.

Exogenous administration of the IDO1 pathway catabolites kynurenine and quinolinic acid led to activation of β-catenin and proliferation of human colon cancer cells, resulting in increased tumor growth in mice [98]. In a similar study, high IDO expression levels in tumor cells were positively correlated with myometrial invasion, nodal metastasis and lymph-vascular space involvement [99]. Further, a significant correlation was detected between high levels of IDO1 expression and reduced numbers of CD3^+^, CD8^+^ and CD57^+^ cells infiltrating both the tumor epithelium and stroma.

Glioblastoma multiforme (GBM) is an aggressive adult brain tumor with a poor prognosis. One hallmark of GBM is the gradual accumulation of immunosuppressive and tumor-promoting CD4^+^ FoxP3+ regulatory T-cells (Tregs) [100,101]. Wainwright and colleagues investigated the role of IDO1 in brain tumors and its impact on Treg recruitment and found that IDO1 expression increased recruitment of immunosuppressive Tregs that lead to tumor outgrowth [101]. In contrast, IDO1 deficiency was shown to decrease Treg recruitment and to enhance T-cell-mediated tumor rejection. These data suggest a critical role for IDO1-mediated immunosuppression in glioma and support the continued investigation of IDO-Treg interactions in the context of the suppression of brain tumor outgrowth. Alternatively, in a study performed by Li *et al.* [102] uncovering a link between IDO and the complement, pharmacologic inhibition of IDO synergized with chemo-radiation therapy to prolong survival in mice bearing intracranial glioblastoma tumors. They showed that pharmacologic or genetic inhibition of IDO allowed chemo-radiation to trigger widespread complement deposition at sites of tumor growth. Chemotherapy treatment alone resulted in collections of perivascular leukocytes within tumors, but no complement deposition. Adding IDO blockade led to upregulation of VCAM-1 on vascular endothelium within the tumor microenvironment, and further, adding radiation in the presence of IDO blockade led to widespread deposition of the complement. Mice genetically deficient in complement component C3 lost all of the synergistic effects of IDO blockade on chemo-radiation-induced survival.

Indoleamine 2, 3-dioxygenase is overexpressed in many different tumor types, including breast cancer [103]. Chen and colleagues have reported the expression of IDO1, estrogen receptor (ER), progesterone receptor (PR), human epithelial receptor 2, cytokeratin 5/6, epithelial growth factor receptor, phosphorylated AKT, neoangiogenesis, nitrogen oxide synthetase 2 (NOS2), cyclooxygenase 2 (COX2), FoxP3, CD8^+^ and CD11b molecules on archival breast cancer tissue [104]. The experimental results showed that IDO1 expression was higher in ER+ tumors compared to ER− tumors. Further, tumor survival was found to be better in ER+ patients.

A connection between elevated urinary tryptophan catabolites and bladder cancer was first reported in the 1950s [105]. Since then, elevated levels of IDO-generated catabolites have been found to be associated with a number of malignancies [94]. This phenomenon was initially thought to be a consequence of IFN-γ treatment, known to stimulate IDO expression in tumor cells [94]. For some time, the significance of IDO promotion of cancer survival was questioned by its observed function in the prevention of allogenic rejection and by the evidence that IDO is overexpressed in most tumors and tumor draining lymph nodes [106,107]. A major question is how does IDO become deregulated in cancer cells? A possible answer is emerging from studies of Bin1, a tumor suppressor gene that is often inactivated during cancer, which seems to inhibit cancer development to a significant extent by limiting immune escape [108]. Studies aimed at understanding how Bin1 restricts tumor outgrowth identified the establishment of immune tolerance through deregulation of IDO1 as a likely explanation [108]. Deletion of the Bin1 gene from mammalian cells resulted in an increased *IDO1* gene expression stimulated by IFN-γ. In this study, *in vitro* transformation of Bin1-null and Bin1-expressing primary mouse embryo keratinocytes with c-myc and mutant Ras oncogenes produced cell lines with similar *in vitro* growth properties. However, when these cells were grafted into syngeneic animals, the Bin1-null cells formed large tumors, whereas the Bin1-expressing cells formed only indolent nodules. Together, these findings suggest that the overexpression of IDO1, which accompanies Bin1 loss, promotes tumorigenicity by enabling immune escape. The attenuation of Bin1 together with IDO overexpression observed in human cancers warrants further evaluation of the relationship between these two metabolic events.

Together, the data suggest that tumors exploit the induction of IDO1 as a dependable mechanism for survival through enhanced suppression of immunity. Recent studies using *ex vivo* antigen-loaded DCs loaded with tumor antigens were shown to improve the immune response to the cancer [109,110]. The goal of recent DC-derived tumor vaccines are to elicit the CD8^+^ T-cell response [111]. However, in order to reach this goal, the DC-elicited adaptive immune response must be able to overcome the immunomodulatory effects of the tumor [109,110,111].

### 4.4. The Role of Indoleamine 2, 3-Dioxygenase in Tissue-Specific Autoimmunity

Organ and tissue-specific autoimmunity requires the initial release of specific autoantigens characteristic of a given tissue or organ that can be recognized by DC pattern recognition receptors (PRR) [59]. The prototypic tissue-specific autoimmune diseases that are presented here include type 1 diabetes (T1D) and multiple sclerosis (MS). Type 1 diabetes is an autoimmune disorder in which auto-reactive T-cells selectively destroy the pancreatic islet insulin-producing beta cells. The genetically diabetes-prone NOD mouse strain is a murine model of human type 1 diabetes. Diabetic NOD mice generally die from the effects of hyperglycemia, reflecting T-cell-mediated destruction of the insulin-producing pancreatic islet β cells. The predisposition of NOD mouse development of autoimmunity may involve defects in the mechanisms of both peripheral and central tolerance [65,112]. Defective in IDO1 expression, NOD mouse pDCs fail to upregulate IDO1 in response to stimuli, such as the pro-inflammatory cytokine IFNγ, one of the most potent inducers of IDO expression and catalytic function [65,113]. Studies conducted by Pallotta *et al.* showed that forced IDO1 expression in dendritic cells rescues both IDO enzymatic and signaling activities, providing substantial proof that global IDO defects predispose NOD mice to autoimmunity [65].

In contrast, multiple sclerosis (MS) is a chronic inflammatory disease of the central nervous system (CNS) associated with an immune reaction against components of the myelin sheath, predominantly myelin basic protein [114]. Experimental autoimmune encephalomyelitis (EAE), the animal equivalent of MS, is a prominent animal model that researchers studying MS use to assess disease progression. Based on their immunosuppressive properties, human mesenchymal stem cells (hMSC) provide a promising tool for cell-based therapies of autoimmune diseases, including MS. Murine MSCs (mMSC) were used to characterize and optimize the route of administration, motility, cellular targets and immunosuppressive mechanisms in mouse models of autoimmune diseases, such as experimental autoimmune encephalomyelitis (EAE) [115]. Tryptophan catabolism by IDO1 is a major endogenous metabolic pathway that tightly regulates immune responses throughout the nervous system. The activity of IDO1 contributes to the immunosuppressive phenotype of hMSC [115]. In a study conducted by Lanz and his colleagues, the authors showed that although IDO1 is inducible in bone marrow-derived mMSC by pro-inflammatory stimuli, such as interferon-γ (IFN-γ) and ligands of toll-like receptors (TLR), disease induction does not lead to catabolism of tryptophan *in vitro* [115]. Thus, IDO1 does not appear to be involved in mMSC-mediated immunosuppression in EAE. While mMSC suppressed the activation of the antigen-specific myelin oligodendrocyte glycoprotein (MOG)-reactive T-cell receptor (TCR) in transgenic Th cells in MSC-T-cell co-cultures, neither pharmacologic inhibition nor genetic ablation of IDO1 reversed this suppressive effect [115]. However, in this study, systemic administration of both IDO1-proficient and phenotypically identical IDO1-deficient mMSC equally resulted in amelioration of EAE. Thus, mMSCs, unlike hMSCs, do not display IDO1-mediated suppression of antigen-specific T-cell responses. During experimental autoimmune encephalomyelitis (EAE), IDO1 induction was shown to downregulate neuro-inflammation [115]. Inhibition of IDO activity by daily subcutaneous administration of the specific IDO inhibitor 1-methyl-DL-tryptophan was shown to significantly exacerbate EAE [116]. Further, cytosolic DNA sensing activates the stimulator of IFN genes (STING) adaptor to induce IFN type I (IFN-αβ) production [116].

Constitutive DNA sensing to induce sustained STING activation incites tolerance breakdown, leading to autoimmunity. In a study conducted by Lemos and colleagues, it was shown that systemic treatments with DNA nanoparticles (DNPs) induced potent immune regulatory responses via STING signaling that suppressed EAE when administered to mice after immunization with myelin oligodendrocyte glycoprotein (MOG), at EAE onset or at peak disease severity. DNP treatments attenuated infiltration of effector T-cells into the CNS and suppressed innate and adaptive immune responses to myelin oligodendrocyte glycoprotein immunization in spleen. Therapeutic responses to DNPs were shown to be critically dependent on IDO enzyme activity in hematopoietic cells. These findings reveal dichotomous roles for the STING/IFN-αβ pathway in either stimulating or suppressing autoimmunity and identify STING-activating reagents as a novel class of immune modulatory drugs [116]. Thus, local expression of IDO during inflammation may be a mechanism for self-protection that limits antigen-specific immune responses in the CNS.

## 5. Immune Suppressive Vaccines: The Case for CTB-Autoantigens and Their Relationship to IDO1

By the avoidance of environmental factors thought to promote autoimmune diseases in genetically at-risk individuals, autoimmune diseases, such as type 1 diabetes, might be eradicated. However, these environmental factors have not as yet been clearly identified and may be ubiquitous. Since the early 1980s, prevention, following disease initiation, has been the focus of attention with many candidate therapeutic agents, mainly immunosuppressive drugs [117,118,119,120]. Prevention is, however, more applicable to early preclinical disease than to recent onset clinical disease, in which pancreatic islet beta cell destruction is more advanced [34,121].

Until the present, prevention of infectious disease by exposing the immune system to a weakened, non-toxic or dead infectious agent has been the traditional method of vaccination [120,121,122]. Prominent among immunological enhancement or adjuvant strategies are the bacterial and plant AB subunit toxins, which include shiga toxin, anthrax toxin, ricin toxin, the heat sensitive enterotoxin from *E. coli* and the cholera toxin CTA and CTB subunits [122]. In contrast to the toxic CTA subunit, the nontoxic CTB subunit displays both carrier and mild immune-stimulatory properties [123]. When linked to pathogen antigens, CTB was shown to impart immune-stimulatory properties that convey increased levels of immune stimulation in response to the linked antigen [124]. However, when CTB is linked to “self” proteins, the result is often enhanced immunological suppression of autoimmunity. Demonstrating the adjuvant capability of the cholera toxin B subunit, the linkage of CTB to an autoantigen (ovalbumin) was shown to provide up to a 10,000-fold reduction in the amount of autoantigen required for generating immune tolerance [59,122,125,126]. In type 1 diabetes, self-proteins, like insulin, become more strongly immunosuppressive when linked to CTB. Oral administration of the CTB subunit coupled with insulin or the GAD_35_ autoantigen was shown to induce immunological tolerance and suppression of type 1 diabetes in NOD mice [125,127].

Additional forms of tissue-specific autoimmunity were capable of being suppressed by CTB-linked autoantigens. Behcet’s disease (BD) is an inflammatory tissue-specific autoimmune disorder characterized by uveitis, oral and genital ulcers, as well as cutaneous, vascular, joint and neurological inflammation [128,129]. Fusion of an uveitogenic HSP60-derived peptide (aa 336–351) with CTB resulted in significant protection against mucosally-induced uveitis and other Behcet’s disease symptoms [130].

Multiple sclerosis (MS) is an inflammatory disease of the central nervous system (CNS) characterized by localized myelin destruction and axonal degeneration [131]. An autoimmune reaction against myelin antigens of the CNS was shown to contribute to the immunopathological mechanisms of MS [132]. Myelin oligodendrocyte glycoprotein (MOG) is a key CNS-specific autoantigen for primary demyelination in multiple sclerosis. Fusion of CTB with myelin oligodendrocyte glycoprotein (MOG) was shown to provide protection against the development of MS symptoms [133].

Type 1 diabetes (T1D) is a well-studied prototypic tissue-specific autoimmune disease resulting from auto-reactive lymphocyte destruction of the pancreatic islet insulin-producing β-cells [34,134,135,136,137]. Progressive loss of islet β-cell function leads to insulin deficiency and high blood glucose levels (hyperglycemia). Increased cellular oxidative stress and chronic inflammation generated by hyperglycemia can result in neural and circulatory complications that lead to amputation, loss of kidney function, blindness, heart attack and stroke, resulting in early mortality [34,138,139]. Linkage of CTB to insulin (CTB-INS) provided a protective effect against the onset of type 1 diabetes in NOD mice [123,126,140]. Initial oral immunization experiments showed that feeding small amounts (2–20 µg) of CTB-INS could effectively suppress β-cell destruction and clinical diabetes in pre-diabetic NOD mice [123,124,141]. Initial recognition of the mechanism underlying vaccine-mediated immune suppression was based on CTB-INS induction of CD4^+^ regulatory T-cells (Tregs) in NOD mice [142]. Demonstrating the broad range of applications of this vaccine strategy for the suppression of autoimmunity, conjugation of CTB with islet auto-antigens, including insulin and glutamic acid decarboxylase (GAD), was shown to induce immunological tolerance through the suppression of human DC maturation [123,127].

### 5.1. Immunosuppressive Vaccine Induction of Indoleamine 2, 3-Dioxygenase

Autoimmune diseases result from misdirected immune attack on one’s organs and tissues and together are responsible for the death of more than 700 million people worldwide annually, generating a public health crisis comparable to heart disease and cancer [59,143]. Approximately 20% (one in five) Americans suffer from terminal autoimmunity because no cure is available [119,143]. About 80% of patients are women with a 2–5-times greater risk of autoimmune disease onset among African, Hispanic and Native American women than those of European descent [119,137,143,144,145]. Most autoimmune diseases are tissue specific and are initiated by specific self-antigens, suggesting a common underlying cause [59]. Dendritic cells (DCs) recognize and process self-antigens and are the first immune cells to surround pancreatic islets, indicating a prominent role in type 1 diabetes development [15,58,59,146,147,148]. Disease onset begins when insulin-presenting DCs bind T-cell receptors of cognate naive T helper cells and guide their differentiation into pro-inflammatory T helper and cytotoxic T-cells that attack the insulin producing islet β-cells [13,149,150,151,152]. Among the most promising therapeutics, immuno-suppressive vaccines were shown to arrest autoimmunity in animals [123,124,141,153,154]. However, vaccine efficacy in patients remains untested, because their mode of action is unknown. Due to the variability of patient responses to individual vaccines, combinatorial vaccines may provide the most effective form of treatment. Multicomponent vaccines composed of the cholera toxin B subunit (CTB) linked to self-antigens were shown to prevent uveitis, multiple sclerosis and type 1 diabetes in animal models of autoimmunity [124,131,132,142,153]. Recent experiments showed that a CTB-insulin vaccine that induced tolerance to diabetes autoantigens in humans is linked to inhibition of DC maturation [153]. While the mechanism responsible for vaccine-induced tolerance remains unknown, analysis of the vaccinated DC proteome revealed dramatic upregulation of the tryptophan catabolic enzyme indoleamine 2, 3-dioxygenase (IDO1) [34]. Emphasizing the enzyme’s role in autoimmunity, increased IDO degradation of tryptophan accompanied DC suppression of arthritis, asthma, hemolytic anemia, multiple sclerosis, systemic lupus erythematosus and type 1 diabetes [116,117,146,154]. Vaccine-induced IDO1 biosynthesis and enzyme activity in human DCs suggest that kynurenines may be important for vaccine suppression of type 1 diabetes autoimmunity [11,34,56,61,65]. Interestingly, fusion of CTB to insulin was found to be essential for the induction of IDO1 biosynthesis, suggesting that vaccine signaling functions may be involved in the suppression of DC activation [34]. 

## 6. Conclusions

Immunological tolerance that occurs in response to IDO1 induction results in the depletion of cellular tryptophan levels and the production of kynurenines that kill pro-inflammatory T-cells and induce the proliferation of immunosuppressive regulatory T-cells. The pivotal role IDO1 plays in immune suppression is dependent on the essential nature of tryptophan and the profound effect tryptophan catabolism has on the activation or inhibition of immunity and cellular metabolism. Thus, regulation of IDO biosynthesis or activity in antigen-presenting cells of the innate immune system is important in the regulation of their responses to immunological insults, such as infection, autoimmunity and cancer. Data presented in this review suggest that adjuvant-autoantigen vaccine-induction of IDO1 biosynthesis is a likely mechanism for specific and effective immune suppression of DC maturation, leading to the induction of durable peripheral tolerance. Understanding how adjuvant-autoantigen vaccines modulate IDO1 activity in human dendritic cells will facilitate improvements in combinatorial vaccine potency and safety, moving this effective immunosuppressive strategy closer to clinical applications for the prevention of autoimmunity and diseases that possess a strong chronic inflammatory component.
